# P-1847. Exploring the correlation between time to positivity and positive culture bottle count in *Staphylococcus aureus* bacteremia: implications for metastatic lesion development

**DOI:** 10.1093/ofid/ofae631.2008

**Published:** 2025-01-29

**Authors:** Kazuhiro Ishikawa, Satoru Sekiya, Nobuyoshi Mori

**Affiliations:** St Luke's international hospital, Chuo-ku, Tokyo, Japan; St Luke's international hospital, Chuo-ku, Tokyo, Japan; St. Luke's International Hospital, Tokyo, Tokyo, Japan

## Abstract

**Background:**

While numerous studies have investigated the correlations among time to positivity (TTP) in blood cultures, the number of positive bottles, and mortality rates, the association with metastatic lesion formation in *Staphylococcus aureus* (*S. aureus*) bacteremia remains poorly understood. This investigation aims to assess the impact on the severity of the, mortality, and the frequency of metastatic lesions.

Study Population Flowchart

**Figure:**
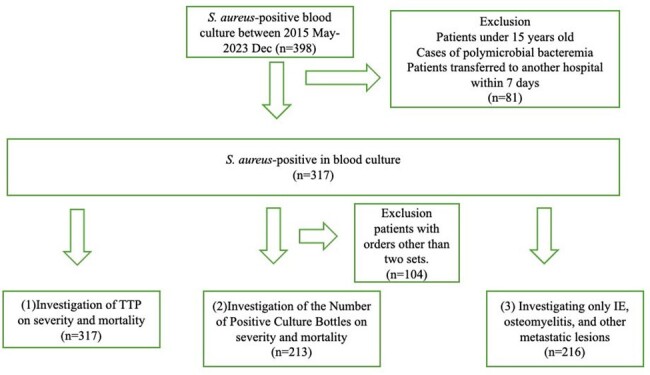

Flowchart illustrating categorization of S. aureus-positive blood culture cases for assessing TTP, bottle count, severity, mortality, and metastatic lesions.

**Methods:**

A retrospective analysis was conducted on patients over 15 years old, admitted to St. Luke's International Hospital between 2015 and 2023, who tested positive for S. aureus in blood cultures. Patients with polymicrobial patterns or transferred within a week were excluded. Metastatic lesions were identified through chart reviews and imaging.

Baseline Characteristics of Study Participants

**Figure:**
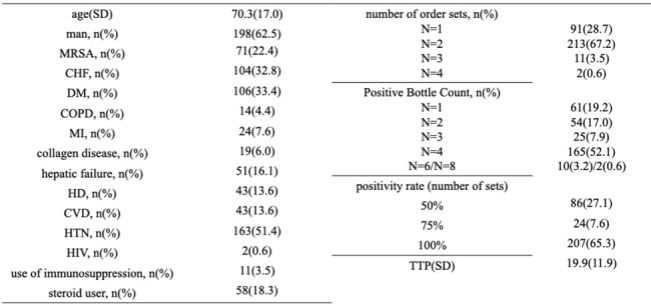

**Results:**

From 398 initial cases, 317 patients were included after exclusions (Figure 1). The patient cohort had an average age of 70.3 years (Standard deviation, SD: 17.0), with 62.5% being male, and 22.4% with MRSA infections. The average TTP was 19.9 hours (SD: 11.9) (Table 1). No significant differences were observed in TTP or bottle count in relation to mortality or severity. Further investigation into the relationship with metastatic lesions shown in Table 2 revealed that TTP for patients with intravascular infections was significantly shorter, averaging 16.81 hours (SD: 5.68), compared to 23.45 hours (SD: 13.84) in bacteremia without metastatic lesions. TTP for bone and joint infections was significantly shorter, averaging 17.88 hours (SD: 8.26), compared to bacteremia without metastatic lesions. SSTI did not show a significant difference in TTP. A weak correlation was found between TTP and metastatic lesion counts (r=0.203) (Figure 2). Additionally, TTP and positive set count showed a weak correlation (r=0.544). When TTP cannot be determined, bottle counts may offer an alternative indicator.

number of complications due to S. aureus

**Figure:**
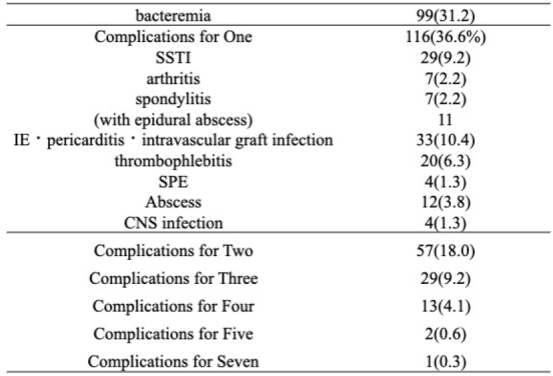

**Conclusion:**

While TTP and bottle count were weakly correlated, they failed to predict severity or mortality in *S. aureus* bacteremia. Positivity within 24 hours warrants further examination for metastatic conditions like intravascular and bone infections. Yet, TTP and bottle counts' predictive value for metastatic lesion quantity is minimal, highlighting the need for thorough physical exams and imaging.

Correlation between number of disseminated lesions and positive time

**Figure:**
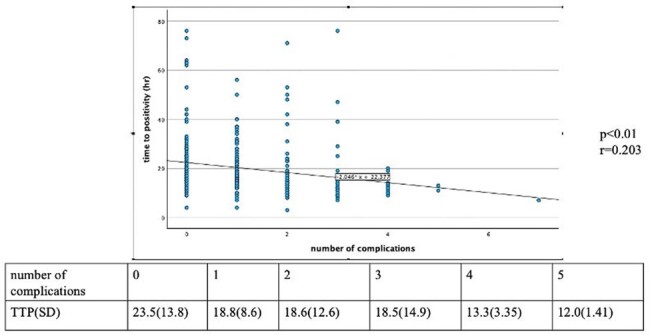

**Disclosures:**

**All Authors**: No reported disclosures

